# Influence of Epoxy Glue Modification on the Adhesion of CFRP Tapes to Concrete Surface

**DOI:** 10.3390/ma14216339

**Published:** 2021-10-23

**Authors:** Andrzej Szewczak

**Affiliations:** Faculty of Civil Engineering and Architecture, Lublin University of Technology, Nadbystrzycka 40, 20-619 Lublin, Poland; a.szewczak@pollub.pl; Tel.: +48-815-384-428

**Keywords:** profilometers, epoxy resins, sonication, viscosity, adhesion, pull-off test, hardness, tensile strength

## Abstract

Strengthening structural concrete, steel or wooden elements with reinforcement tapes is currently a popular method of extending the durability of buildings. In the glued joint Carbon Fibre Reinforced Polymer (CFRP) tape—concrete substrate, the most critical element is the adhesive layer connecting both materials. The glued joint participates in the transfer of stresses between the reinforced element and the reinforcement tape. Among the numerous analyses of this type of joint, the work resulting from the action of shear stresses (shearing) is considered most frequently, which also results from the originally developed computational models emerging with the development of research on the processes of adhesive effectiveness. The subsequent theories considered the share of other stresses, which is also related to the complex nature of the phenomenon of glue adhesion on various surfaces. Research shows the possibility of modifying the adhesion of the glue by altering its composition and the target surface of application. The study contains the results of research on the possibility of changing the adhesion of the glue to a concrete surface prepared by grinding and sandblasting. The selected epoxy resin has been modified by using the additives of microsilica and carbon nanotubes. Effective mixing of ingredients was achieved due to the use of sonication in the mixing process. Then, the adhesives prepared in this way were used to stick fragments of CFRP tape to concrete surfaces: cleaned, ground and sandblasted. A modified version of the pull-off test was used to determine the effectiveness of adhesion the CFRP tapes to concrete. The results are the final stage summarizing a series of studies including other parameters affecting the bonding efficiency and durability of adhesive bonds.

## 1. Introduction

**Figure 1:**
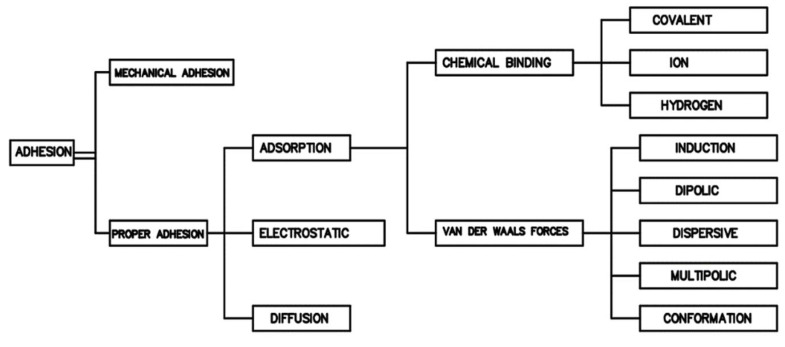
Adhesion types and models (based on [3,4,6]).

In physicochemistry, adhesion is one of the most difficult phenomena to define unambiguously. According to [1,2,3,4,5], it is a series of phenomena that may occur at the contact of surfaces of adjacent materials. In Latin, *adhesio* means adhering, sticking [3]. Nevertheless, such a general definition explains this phenomenon to a very small extent. The main reason that makes it impossible to define adhesion in an unambiguous way is its very complex nature. This is due to the fact that adhesion depends on many factors [2,3,6]. Importantly, the individual definitions complement each other. Over the years, the research on adhesion, the development of which was possible owing to the development of the field of science, the so-called surface physicochemistry [1,2,7], have resulted in an increasingly consistent but also multi-directional definition of adhesion [4,5,8]. The definition of McBain and Hopkins [9] was developed as one of the first. It was based on an adhesion model showing the mutual interlocking of two surfaces. Further analyses, the development of new research methods and a more detailed understanding of adhesion allowed extending this theory. Hence, several models of adhesion are adopted in the literature, along with the main factors determining them. The most common division in the literature is presented in Figure 1 [3,4,6].

As can be seen from the diagram, apart from the original (first developed mechanical model), which depends mainly on the interlocking of rough surfaces of the materials to be joined [2,4], other factors also have an influence on the adhesion. The proper adhesion depends primarily on the physico-mechanical interactions, in particular the possible occurrence of chemical bonds (according to the types shown in the diagram) between the atoms located on adjacent surfaces. The so-called adsorption theory takes into account chemical bonds, but depending on the conditions and method of joining materials as well as their chemical composition, individual types of chemical bonds do not have to occur together at the same time [3,10,11,12,13]. Both classic chemical bonds resulting from chemical reactions, as well as less durable van der Waals interactions—essential for adhesion—are important [14]. Nevertheless, it is believed that the adsorption theory best describes the adhesion resulting from intermolecular interactions [1,3,15,16]. 

Other adhesion mechanisms most frequently described in the literature are: electrostatic adhesion resulting from the interactions of charges at the interface [3,4,17], diffusion consisting in joining materials as a result of mutual passage and permeation of charges between surface layers [2,18,19], the theory of weak boundary layers taking into account the possibility of the occurrence of factors weakening adhesion on the joined surfaces, i.e., surface pores, impurities, voids [2,3,4,20] and the acid-base theory described in [2,3,4,21] relating to the acceptor—donor capacity of chemical compounds [2,15,21].

Gluing building materials is a commonly used method of joining them, largely based on adhesion and cohesion [22,23,24,25]. Modern technology and research on material engineering make it possible to obtain the adhesives that allow for joining many types of materials. Numerous publications and papers present the results of research on the possibility of shaping, making and using glued joints in a wide range of applications, also in relation to numerical modeling [3,26,27,28,29,30,31,32]. Among many factors determining the durability of a glued joint, the most important are:

- Method of surface preparation—methods based on mechanical, physical or chemical processing are mainly considered [3,33,34];
- Type of materials to be joined—each material is characterized by different physical, mechanical and chemical properties, which influences the formation of particular types of adhesion; adhesives may react with the substrate to form chemical bonds (adsorption model) or only fill in surface irregularities (mechanical model) [3,23,35];
- Type of adhesive used—the polymer adhesives based on resins (epoxy, phenolic, polyester, polyvinyl, polyacrylic, phthalic, polyurethane, amine) are most frequently used [24,36,37,38];
- The working conditions of the glued joint, e.g., temperature, humidity, static or dynamic load [39,40];
- Mechanical parameters of the adhesive and the substrate, depending on the expected working conditions and the type of joint [40,41,42];
- The method of glue preparation and its composition—one-component, two-component, chemically or temperature-cured adhesives are distinguished [24];
- Surface roughness—defined as the distribution of unevenness, cavities, pores, scratches on the surface of the intended use of the adhesive; roughness describes the contact surface, affects the penetration depth of the adhesive; the methods of surface preparation described in the first section focus to a large extent on the development of the specific surface by differentiating the topography of the substrate [3,34,43,44,45].

**Figure 2:**
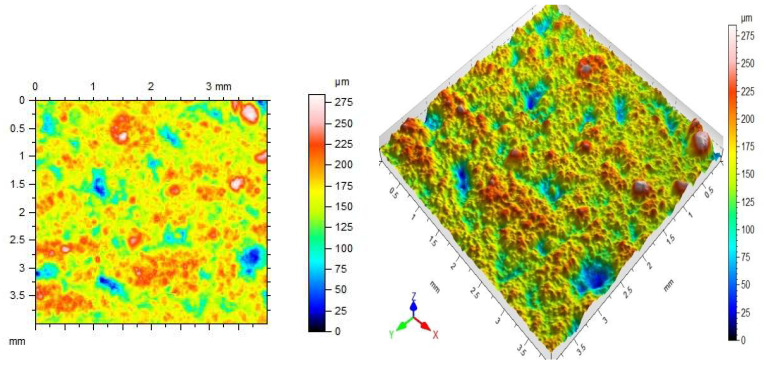
Sample 2D and 3D profilogram of the ceramic surface.

Regarding the last point, there are different methods of roughness testing. One of the most frequently used methods is contact profilometry [46,47,48]. During the examination, the image is obtained as a result of the needle moving over the tested surface. A needle with a diameter and radius appropriately selected for the unevenness of the substrate can be moved vertically and is connected to a module recording the value of displacement received as optical or electrical signals. In this way, the software creates an image of the surface, an example of which is shown in Figure 2.

Glued joints are analyzed in several ways. The vast majority of tests, due to the high load capacity of these connections [27,32,49,50,51], focus on determining the strength, load capacity or durability of such connections in the tangential load scheme (tangential stresses). Their value reaches the maximum at the edge of the joint and can be adjusted e.g., the use of an appropriate flow of glue in a lap or overlap joint [27,52,53]. Moreover, bending, tearing or tearing strength analyses are carried out [3,50]. The development of research has resulted in the form of computational models for glued joints. The simplest of them, e.g., the Goland, Reisner or Volkersen models ignored the stresses originating from reasons other than the shear of the weld, mainly normal stresses [27,54,55]. Moreover, the distribution of stresses along the thickness of the weld was not taken into account, which classifies these theories as linear-elastic. The further development of research on linear-plastic models has resulted in, e.g., the Grimes and Greiman models [27,56]. Despite employing some simplifications, they reflected the real state of stresses in the glue joint in a much better way. The exact state effect was obtained after introducing three-dimensional analyses, also with the use of the finite element method [31,57,58]. Nevertheless, further research and analyses are being carried out on the best simulation of glued joints and taking into account the results of these studies in computer analyses, as the complicated and multidirectional description of the theory of adhesion leads to the creation of new theories and models, which are better also from the point of view of designing such connections. In [59,60,61], the authors focus on a very advanced mechanism of molecular dynamics, based on complex computer analyses of intermolecular interactions. Accurate analyses of the molecules’ movements, both of the predictable and probable nature, must take into account, e.g., the stress states in the glued joint subjected to not only mechanical loads but also environmental factors. It allows for a full explanation of the physicochemical phenomena occurring in the solid phase. The three-dimensional simulation of the chemical molecule behavior that polymer compounds are built on is possible thanks to the use of equations of the state of atoms motion and combining them in a comprehensive manner with, e.g., Newton’s equations of dynamics.

Changing the purpose of the existing buildings, and thus changing the loads affecting them, necessitates looking for the ways to strengthen structural elements such as beams, columns, slabs and foundations [27,59,62,63,64]. These activities are aimed at extending the life of buildings and other structures as well as their durability. Strengthening structural elements also occurs in emergency situations, e.g., when the structure loses its load-bearing capacity as a result of design or execution errors and thus threatens the safety of users [65,66]. A way to increase the load capacity and durability of the elements included in the entire structure is the use of polymer composites, mainly [67]:

Glass fiber—GFRP;

Carbon fiber—CFRP;

Aramid fiber—AFRP;

Basalt fiber—BFRP.

In materials of this type, a suitable type of fiber (glass, carbon, basalt, aramid) constitutes the reinforcement of a polymer matrix, usually made of epoxy, polyester and other polymers. In the case of CFRP tapes, it is also possible to use other fillers for their production. The most important advantages of reinforcing composites are: favorable strength to weight ratio, high tensile strength (see Table 1), high modulus of elasticity and chemical resistance. Reinforcing tapes make it possible to significantly increase the load-bearing capacity of structural elements, which are most often reinforced concrete and steel beams, columns, foundations, walls, vaults or slab ceilings [65,66,68,69].

The research on adhesives (adhesive polymers) is currently focused on two main directions. The first involves obtaining new polymers, mainly by modifying the polymerization reaction and obtaining polymers containing various functional groups, i.e., ester, aldehyde, epoxy, phenolic, amide, carboxyl, vinyl, benzyl, hydroxyl, nitro and amine [37,38]. Combinations of polymers composed of different mers enable obtaining new compounds. In addition, the modifications also include cross-linking and hardening reactions of resins (adhesives). The second trend in the chemistry of polymers, not only adhesives, is modifying their properties through additives with the use of powder fillers [51,70,71]. Their use may significantly affect the functional, mechanical, physico-chemical and rheological properties of the polymer. Additionally, their use makes it possible to limit the amount of polymer obtained most often as a product of refining and processing of crude oil or by synthesizing other mers, which can be a very costly process. The most commonly used powder fillers include: ground limestone and dolomite, chalk, clay from ground brick and other ceramic materials, microsilica, quartz flour, granite and basalt flour, gypsum, mica and soot [7,70,72]. Their dosage is strictly dependent on the required final properties of the obtained adhesive. Depending on the chemical nature of the filler molecules, it is possible to activate it in the bulk of the adhesive and create additional chemical bonds [23,70]. The electrons in the filler atoms can also affect the orientation of the functional groups during cross-linking and bonding of the adhesive to the substrate. This fact is of particular importance when taking into account the aging of the polymer layer as a result of changes in its internal structure with the time of its use, consisting mainly in the relocation of electrons and the weakening of chemical bonds between molecules [7,23,37,73]. Other physical parameters of the fillers —shape, size and specific surface of the grains as well as density—allow for increased adhesion of the glue to the target surface.

However, in the case of filler additives, the correct mixing of the adhesive and the filler may be an issue. This is due to the presence of different phases during mixing (polymer as liquid phase, filler as solid phase); differences in density, nature and chemical composition; and filler reactivity [74,75,76]. One of the effective methods of correct mixing of these phases is sonication, i.e., the action of ultrasounds. As shown in the research [75,77], ultrasounds cause a number of phenomena related to ultrasonic cavitation (change of pressure and temperature of the polymer medium, activation of filler molecules constituting the so-called cavitation centers and chemical sonoreactions), which lead to the dispersion of filler molecules, their partial incorporation into the chemical composition of polymer chains and obtaining a homogeneous polymer structure with a filler in the liquid phase [37,74,78]. The phenomena occurring during sonication also affect the distribution of polymer and filler particles after the resin curing process. This means that the sonication process can also be considered a type of adhesive modification and, with appropriate sonication parameters, may lead to changes in the distance of chemical molecules and thus the internal structure of the polymer [79].

After the glue is fixed on the surface of the substrate (glue curing), the durability of the glued joint over time largely depends on the mechanical parameters of the glue. The strength and modulus of elasticity determine the load-bearing capacity of the joint under the loads occurring, e.g., in a reinforced structure or acting on glued elements made of different materials [32,65]. The working time of such a connection, and therefore its durability, is usually assumed for a period of several or even several dozen years. This means that the prospective improvement of these parameters as a result of adhesive modification with the use of fillers is adequately justified.

The aim of the research presented in the article was to determine the effectiveness of the gluing process on three types of surfaces. The selected epoxy adhesive has been appropriately modified with the use of two types of fillers: microsilica and carbon nanotubes. Ultrasounds were the method that allowed the fillers to be effectively mixed with the adhesive. The tear-off strength of CFRP tapes from the concrete surface was adopted as an indicator of the effectiveness of gluing with the modified epoxy resin. A modified version of the pull-off test was used for this purpose. The main reason for taking up the research was the lack of a wider recognition of epoxy resins modification with the described fillers and sonication for changing properties of the adhesives used for gluing CFRP tapes on various concrete surfaces. The research most often is focused separately on the analysis of the influence of filler addition on the rheological and mechanical polymer properties. Analyses of adhesives’ adhesion to various materials are focused mainly on shear or torn glued joints. The conducted research allowed determining the changes taking place in the epoxy resin structure as a result of the use of microsilica and carbon nanotubes. The analyses were the final stage of the issues which were examined and described earlier in [74,75,76,77].

## 2. Materials and Methods

### 2.1. Components and Used Mixtures

Epidian 52 epoxy resin(Ciech Sarzyna, Nowa Sarzyna, Poland) was employed in the research, which is used as a binder or adhesive to connect various materials. In the described tests, the adhesive was applied to stick pieces of CFRP tape to the concrete surface. The adhesive is pure epoxy resin, the parameters of which are presented in Table 2. A total of 12 series of samples were tested, each with 8 readings from the pull-off device. For each resin recipe, series were made according to the following scheme: series of unmodified resin, series of the resin subjected to ultrasounds in the sonication process, series of the resin subjected to sonication with 0.5% microsilica in relation to the weight of the resin and series of the resin subjected to sonication with 0.1% carbon nanotubes in relation to the weight of the resin. The following were used as fillers:

- BASF microsilica (BASF, Ludwigshafen, Germany), with a density of 2.2 g/cm^3^, mean particle size of 0.1 µm and a specific surface area of 20,000 m^2^/kg.
- NanocylTM NC7000 carbon nanotubes by (NANOCYL, Sambreville, Belgium) with a density of 1.3–1.4 g/cm^3^, an average diameter of about 9.5 nm, a length of 1.5 μm and a specific surface area of 250–300 m^2^/g.

The recipes and designations of each series of samples are presented in Table 3.

In order to ensure an appropriate course of the cross-linking reaction, the amine hardener Z1 (triethyltetraamine), (Ciech Sarzyna, Nowa Sarzyna, Poland)was used, the density of which at 22 °C is 0.98 g/cm^3^ and the viscosity is within the value of 20–30 mPa∙s. The amount of hardener followed the recommendations of the Epidian 52 resin manufacturer and was related to the weight of the resin sample before curing.

Before the resin was subjected to modifications, the density and viscosity of unmodified resins were established as reference values and factors that may determine the final adhesion of the adhesive to the concrete surface. Then, the series ER52/S, ER52/S/M, ER52/S/N were subjected to the sonication process, which lasted 3 min. This time was determined at the stage of preliminary tests as the most optimal with regard to the necessity of mixing the fillers in the resin. During sonication, the processes related to the rapid mixing of the resin mass, carbon nanotubes and microsilica were observed. The mixing of the fillers with the adhesive was possible mainly due to the ultrasonic cavitation phenomenon, which was advantageous in this particular case. In the case of the ER52/S series, it was assumed that the impact of ultrasounds is a modification method in itself, according to [74,76]. The ultrasounds were generated by means of a UP 400S stationary sonicator (Hielscher Ultrasonics GmbH, Teltow, Germany), emitting waves with a frequency of 24 kHz with an adjustable power range from 0 to 400 W, and with a cycle (amplitude) adjustment in the range of 0.5–1. After the sonication ceased, the viscosity and temperature of the resins were measured again—the action of ultrasounds caused the temperature of the polymers to increase, thus reducing their viscosity. In the final stage of preparing the adhesives for curing, their viscosity was measured again after the temperature returned to its original value of 22 °C. The results of these measurements were presented in [76].

Concrete class C30/37 was designed to determine the adhesion of glues to concrete. The concrete itself was not analyzed; the compressive strength (determined on 5 cubic samples with edge length of 15 cm) and the modulus of elasticity after 28 days (3 cylindrical samples with a diameter of 15 cm and a height of 30 cm) were tested only to confirm the correctness of the design assumptions made when selecting the concrete recipe. A mixture of granite aggregate and sand was used with a sand point equal to 30%, CEM I 42.5R Portland cement. The following amounts of ingredients were used to prepare 115 L of samples:

- Granite—165.39 kg;
- Sand—44.84 kg;
- Cement—45.23 kg;
- Water—20.18 kg.

**Figure 3:**
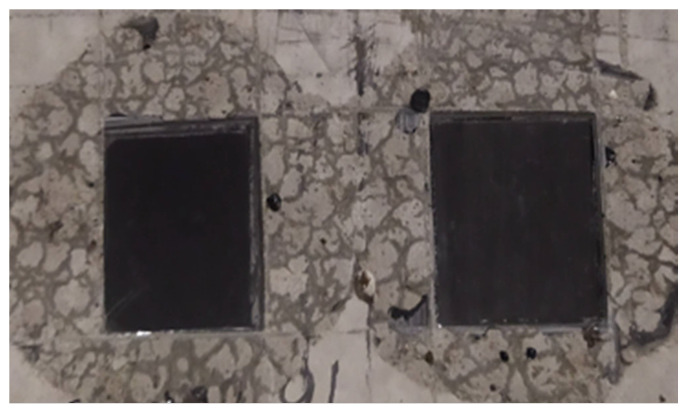
View of the CFRP tape pieces glued to the cleaned surface (ER52 series).

During maturation, the samples were stored in a water bath. After 28 days, they were dried, and their mechanical parameters were examined according to [80]. Then, the adhesion test of 3.0 × 2.5 cm CFRP tape pieces (Figure 3) was carried out on identical samples that were not used when determining the strength class. For this purpose, 1.2 mm thick Sika CarboDur S CFRP tape (Sika Poland, Warsaw, Poland) was used. The samples were glued to the surfaces of the cubes, which were in contact with the walls of the mold (Figure 3). The following methods of concrete surface preparation were selected for the tests:

- The concrete surface is cleaned of dust, concrete and concrete milk;
- Concrete surface polished with a diamond disc;
- Concrete surface wet sandblasted with quartz sand with a diameter of 0.1–0.5 mm.

### 2.2. Methodology

The research program included the following tests:

- Measurement of the viscosity of the unmodified resin * [77];
- Measurement of the temperature and viscosity of the resin when the sonicator is turned off *;
- Measurement of the temperature and viscosity of the resin when the resin reaches the output temperature of 22 °C *;
- Measurement of surface free energy (SFE) on the surface of cured resins * [77];
- Measurement of resin hardness *;
- Measurement of the tensile strength of the resin *;
- Measurement of the elastic modulus and Poisson’s ratio of the resin * [76];
- Making concrete samples of class C30/37 constituting the base for sticking fragments of CFRP tape with the selected glue;
- Preparation of the sample surface according to the scheme presented in point 2.1.;
- Sticking the tape to the samples;
- Determination of profilometric parameters for a cleaned, ground and sandblasted concrete surface;
- Testing the adhesion of samples to the concrete substrate using the pull-off test;
- Analysis of the obtained results and their reference to other results described in [75,76,77].

* Test described in other articles by the author.

#### 2.2.1. Physical Properties

As it was already mentioned, during the tests, a total of 3 measurements of the resin viscosity were carried out for each recipe—the viscosity of the adhesives before modification, the viscosity of the adhesives prepared according to the recipes presented in Table 3 at the moment of switching off the sonicator and the viscosity when the temperature of the resins returned to the comparative value of 22 °C. An H-type rotary stationary viscometer (FungiLab, Barcelona, Spain), equipped with an R2 spindle, and a PT—105 laboratory thermometer by Elmetron (Zabrze, Poland) were used for this purpose. During the viscosity test, the speed of 100 rpm was assumed for all recipes. The accuracy of the viscosity measurement is 0.1 mPa·s, and the temperature 0.1 °C.

#### 2.2.2. Surface Properties

**Figure 4:**
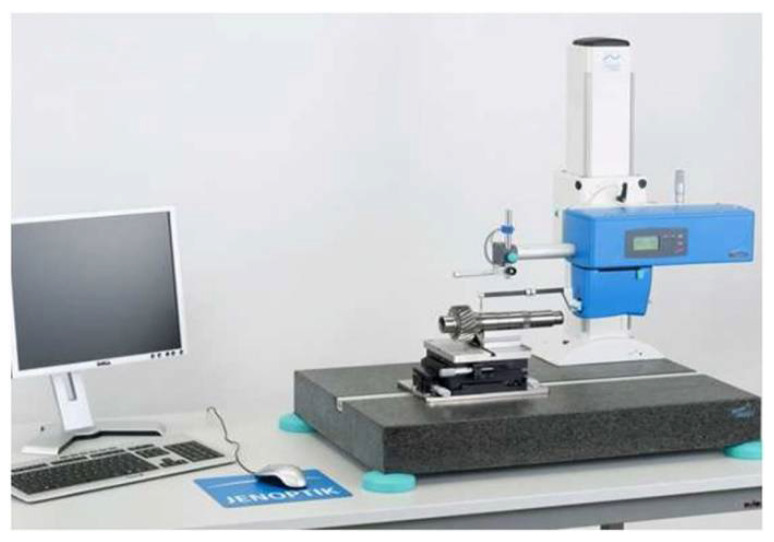
Test stand for profilometric testing.

For the concrete surfaces prepared according to the described scheme, the roughness parameters were tested using the T8000 RC120-400 contact profilometer by Hommel-Etamic (Charlotte, NC, USA) (Figure 4). The measurements were carried out on 5 × 5 cm area sections. Each measurement consisted of 48 movements of the profilometer over the surface of the samples. Owing to the built-in control module and the profilometer which records the movement of the needle on the surface of the samples, three-dimensional images of the surfaces prepared in this way were obtained for comparison. It was assumed that when the roughness and specific surface—which is filled by the adhesive while sticking the tape fragments—are differentiated, the results of adhesion of the samples to concrete will also vary. The results of these analyses will be presented in a further part of the article.

#### 2.2.3. Mechanical Properties

The concrete samples of C30/37 class were tested for compressive strength and average elasticity modulus in order to confirm the requirements of the strength class, in accordance with the recommendations of the standards. Five cubic samples with the edge length of 15 cm were prepared and tested in a CONTROLS testing machine (Milan, Italy) with a load range of 0–3000 kN. The modulus of elasticity was determined in a WalterBai machine (Lohningen, Switzerland) with an attachment containing an electrofusion strain gauge and with a programmed test. 

**Figure 5:**
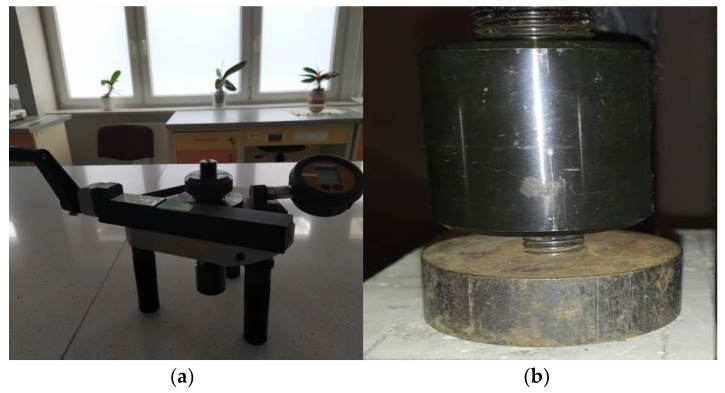
Pull-off testing device (**a**) and the setup of device with the specimen (**b**).

The adhesion of CFRP tape samples to the concrete was investigated using a Dynatest pull-off tester (Gainesville, FL, USA) with a load range of 0–25 kN (Figure 5a,b). Eight measurements were performed for each recipe. The test was carried out within 14 days of sticking the samples. The average thickness of the adhesive layer between the CFRP tape and the concrete surface was 0.5–0.6 mm. The samples were glued to concrete at the age of 360–400 days. The age of concrete at the time of testing resulted from the adopted assumption regarding the practical aspect of strengthening structural concrete and reinforced concrete elements. According to general rules, the elements for which load-bearing capacity has been weakened as a result of exceeding the permissible stresses are subject to strengthening. Strengthening may also be carried out in order to increase the load-bearing capacity when the purpose of the structure is changed. Therefore, fresh concrete is not subject to strengthening, e.g., 28 days after molding. The adopted assumptions were intended to partially refer to the actual conditions for strengthening this type of elements. The peel stress was determined as the quotient of the peel force and the surface area of the CFRP tape sample. It is worth noting that all the conducted studies required strictly defined humidity and temperature conditions and a high level of accuracy.

## 3. Results and Discussion

### 3.1. Physical Properties

#### Viscosity in Unmodified and Modified Resin

**Figure 6:**
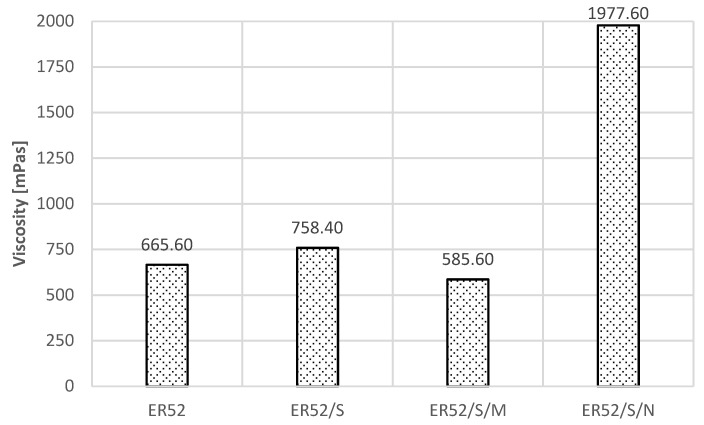
Results of viscosity measurements of unmodified and modified resins (own study, [75]).

The viscosity of resins for individual recipes is shown in Figure 6 [75].

The sonication process decreased the viscosity for the ER52/S/M formulation by approximately 13%, and resulted in 14% an increase for the ER/S formulation as well as a threefold increase for ER52/S/N. As noted in [74,75,76,77], this is the result of the phenomena occurring at the moment of sonicator operation and dynamic mixing of the resin mass and filler particles. Importantly, the shape and chemical composition of the filler molecules are relevant. The explanation of these dependencies requires a direct reference to the chemical structure of resins as polymers and the processes taking place during sonication, which are described in detail in [74,77,81]. In atomic terms, resins are organic compounds of carbon, oxygen and hydrogen. The mers included in the polymer chains contain epoxy functional groups and other hydrocarbon molecules, which undergo initial reorganization under the influence of ultrasounds. As a result of the radical polymerization [37], so-called free radicals are created, i.e., atoms or molecules in an excited state characterized by an increased possibility of forming permanent or temporary chemical bonds, also with filler molecules. In the studies described in [82], it was stated that in such situations, the variable power of the applied ultrasounds, the speed of their propagation in the medium (and therefore also the density of the medium) as well as their amplitude (frequency) may be significant. Resting polymers are characterized by bonding into spherical forms [1,7,37]. When ultrasounds are applied, the spheres expand first, which is also related to the occurrence of Brownian motion. Then, larger conglomerates divide, while dynamic vibrations (cavitation oscillation) cause a local increase in the temperature and pressure of the medium. This causes viscosity, which has also been demonstrated and confirmed in [83,84]. In addition, as already mentioned and described, as a result of ultrasonic cavitation [79,81], microbubbles, containing gas vapors, mainly oxygen, nitrogen and polymer hydrocarbons, are formed. They undergo a very quick formation and collapse due to pressure changes; additionally, they reduce the viscosity during sonication. When the molecules return to the state of physical equilibrium and the initial temperature, they re-create an ordered and compact structure. Microsilica particles form temporary bonds mainly due to the Van der Waals and London forces, which was also demonstrated in [85]. These reactions lead to a reduction in viscosity. It was described in [74] that this may be caused by the process of sharing electrons from microsilica and the electron cloud of the polymer excited during sonication. As the silicon contained in microsilica has the same number of valence electrons as carbon, i.e., four, it has been shown that it is possible to temporarily attach silicon particles to mers, with simultaneous permanent or temporary relocation of oxygen and hydrogen atoms. There is also a partial joining of the free ends of the elements of the polymer chain. In the case of carbon nanotube particles, the increase in viscosity follows a different mechanism. As described in [74], nanotubes are three-dimensional structures in which single chemical bonds dominate between the carbon atoms. Nevertheless, double bonds also appear regularly, but much less frequently. During sonication, these bonds can break. Due to the branched structure of nanotubes resembling a polymer structure and their chemical compatibility with the main polymer building block, which is carbon C, the broken chemical bonds can combine with free radicals derived from the polymer chain. As a result, interpenetrating networks are formed, the structure of the polymer is significantly densified, the free spaces between the mers are filled and the viscosity increases substantially, as shown in Figure 6. Importantly, the addition of microsilica and carbon nanotubes did not affect the gelation and hardening time of the resin after the hardener addition. The analysis of the viscosity results led to the conclusion that the viscosity of the glue modified before hardening directly affects the so-called initial and final adhesion of the glue to the substrate. This is also confirmed by the conclusions included in the research in [85]. The application of ultrasounds at the time of gluing may result in the formation of new connections between the substrate and the carbon fiber tape, mainly by favorable changes in the viscosity and surface tension of the resin and the activation of the original polymer structure.

### 3.2. Surface Properties

**Figure 7:**
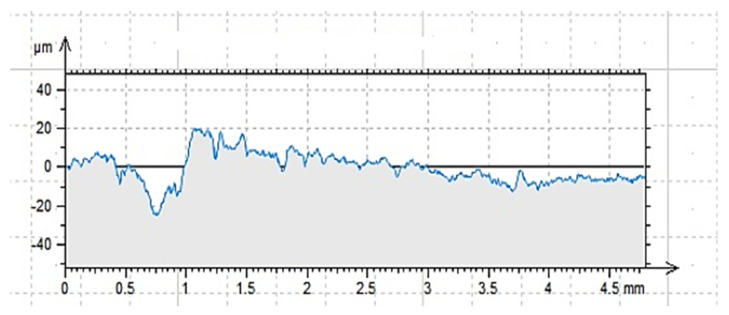
Profilogram of the cleaned surface C, length = 4.80 mm, Pt = 44.7 μm, Scale = 100.00 μm.

**Figure 8:**
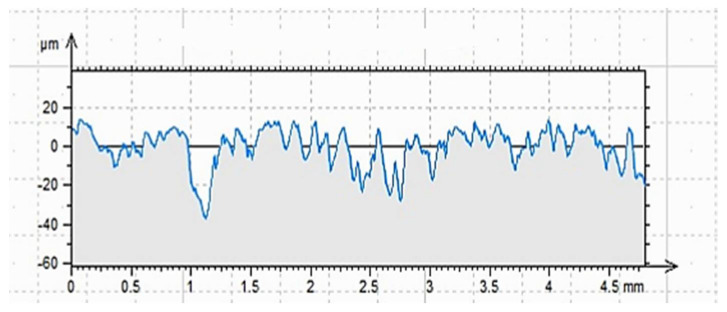
Profilogram of the ground surface G, length = 4.80 mm, Pt = 51.0 μm, Scale = 100.00 μm.

**Figure 9:**
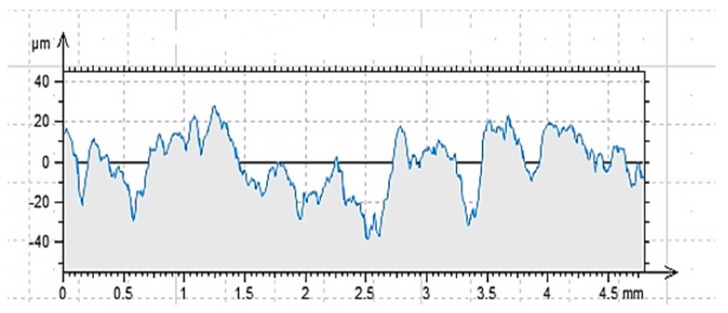
Profilogram of the sandblasted surface S, length = 4.80 mm, Pt = 66.6 μm, Scale = 100.00 μm.

The roughness profiles determined for the cleaned (C), ground (G) and sand-blasted (S) surfaces are shown in Figure 7, Figure 8 and Figure 9 and in Table 4. The following parameters were adopted as comparative factors for individual profiles [86]:

R_p_—maximum height of the roughness profile convexities [μm];

R_v_—maximum depth of the roughness profile recesses [μm];

R_z_—maximum roughness profile height [μm];

R_c_—the difference in the height of the roughness profile part [μm];

R_t_—total roughness profile height [μm];

R_a_—arithmetic mean deviation of the roughness profile ordinates from the mean line [μm];

R_q_—mean square deviation of the ordinate roughness profiles [μm];

R_sk_—roughness profile asymmetry coefficient;

R_ku_—roughness profile slope coefficient;

R_sm_—mean width of the roughness profile grooves.

As indicated by the results of contact profilometry, the properties of the roughness profile are highly diversified for the selected methods of surface preparation. These dependencies are often included in the analysis of glued metal joints and in the issues related to the processing of materials [3,34]. Figure 7, Figure 8 and Figure 9 show clear differences in the shaped profiles. The profile of the cleaned surface, not subjected to additional mechanical treatment, is the least diverse. It is characterized by both the smallest maximum height of the rise and the depth of the recess. Moreover, the mean deviation of roughness unevenness from the mean line is clearly small. This fact is closely related to the conditions of sample preparation. The cleaned surface is relatively smooth, which results from its adherence to the mold wall in which the concrete sample was made. A certain degree of roughness is due to the removal of dirt and cement laitance from the surface of the sample. In this case, the very surface structure of concrete is also important, as it is characterized by natural unevenness, surface pores, microcracks as well as recesses and convexities resulting from the presence of aggregate grains under the outer, top layer of the cement paste, which is described in [4,87,88]. However, this does not mean that the surface does not meet the gluing conditions. It is worth noting that the unevenness of the surface and the adhesive viscosity affect the wettability as well as the ability of the adhesive to cover a part of the surface. In this case, the viscosity of the adhesive is of great importance as it affects the possibility of the resin penetrating the cavities, which was demonstrated in [74,76,89]. In the case of the ground surface, a more varied and rougher surface was obtained. The arithmetic mean of the absolute ordinates is about twice that of the cleaned area. It also results directly from the values of R_p_ (increase by 79%) and R_v_ (increase by 116%). In this case, as shown in Figure 8, the number of recesses is much greater than the line marking the average profile height. The R_z_ value, which is the distance between the highest and lowest point of the profile, is also about twice greater than in the case of the cleaned surface only. This means a larger specific surface area in contact with the adhesive or other materials. It is also confirmed by the almost four-fold higher coefficient of asymmetry of the roughness profile. Such an arrangement of the roughness profile enables better penetration of the adhesive into the unevenness of the substrate and the creation of a better bond resulting from mechanical adhesion. Other adhesion mechanisms are also important. Grinding, as a method of processing, enables the exposure of aggregate grains, in this case granite and sand [3,34,77]. Some of the unremoved cement slurry also remains on the surface. As a result, it is possible to develop more chemical bonds (permanent and temporary) between the adhesive and the substrate than in the case of a cleaned or sandblasted surface. Granite aggregate and sand are characterized by the content of silica in their structure. The grinding process enables the concrete surface to be chemically activated, but only to a limited extent. As has been noted [90,91], grinding allows for obtaining an even surface which is also strong. Simultaneously, according to the research described in [92,93,94], grinding allows for removing the so-called weak boundary layers; however, it is not the most effective method of expanding a concrete surface. Nevertheless, subsequent tests were to confirm the thesis which assumed increased adhesion of modified resins to the substrate prepared in this way. In this case, the effect of ultrasounds is also important, as owing to the reorganization and unfolding of polymer chains, it can cause the connection of free ends with molecules in the surface layer of the substrate [74,76]. Additionally, comparing Figure 7 and Figure 8, one can notice a more even and symmetrical distribution of inequalities. Interestingly, the average width of the grooves, i.e., the distance between the walls of the profile unevenness, is only 3% greater than in the case of the cleaned surface profile. It is probably related to the essence of the grinding process, because of which the finished surface, although rougher, does not have such a variety in this respect. This fact may result in a diversified effectiveness of the adhesive filling the rough surface and covering the unevenness, closely dependent on the adhesive viscosity, the method of its application and processing. The analysis of Figure 9 and the values related to the sandblasted surface profile leads to drawing similar conclusions, but as shown by the results in Table 3, the sandblasting process itself allows for greater deviations from the average profile height value. The R_p_ value is 220% and 123% higher than for the properly cleaned and sandblasted surface, respectively. In turn, the R_v_ value increases by 267% and 123%, respectively. The arithmetic mean of deviations from the average level differs by 251% and 123%, respectively, which also proves the highest specific surface area and the degree of surface differentiation. The roughness profile image shows distinct recesses and convexities, and the distribution is not so uniform compared to the ground surface. The distances between the walls of the cavities are much larger, which is also confirmed by the R_sm_ value that is approximately twice as large. Similar relationships were reported in [3,90,92,93,95,96]. This means that the penetration of the glue into unevenness and recesses is facilitated in the case of a surface prepared in such a way, and the glue also increases the wettability of the walls of the recesses. Sandblasting removes only part of the cement slurry and impurities from the prepared surface, but does not reveal fine aggregate [92,95,97]. Depending on the size of the abrasive grains and the speed of their incidence on the treated surface, a surface with a very good roughness and specific surface is obtained. However, as already mentioned, the possibility of partial surface activation with such preparation can be found by examining the actual adhesion of the glue to the substrate [3,34,52].

### 3.3. Mechanical Properties

#### Compressive Strength of Concrete and Modulus of Elasticity

The results of the compressive strength test, determined for five samples after 28 days of maturation, are presented in Table 5 and Table 6, according to requirements [80]. Owing to the analysis of the results and checking according to the criteria presented in Table 6, it was found that the prepared concrete meets the requirements of class C30/37. 

The value of the mean modulus of elasticity was 31.72 GPa.

The final stage of the research was to look for the correlation between the adopted adhesive modifications and the adhesion of CFRP tape fragments to the concrete surface. The results of measurements using the pull-off method are presented in Table 7, Table 8 and Table 9.

The obtained results were compared with those for tensile strength and surface hardness of resins after hardening, described earlier in [77] and presented in Table 10, which were performed at an earlier stage of the research. As can be seen, the highest strength value, 15% higher, was achieved for the samples modified with sonication. The strengths of the ER52/S/M and ER52/S/N series were about 20%, on average. It is also worth noting that all series were characterized by a similar surface hardness value. This may be suggested by the conclusion also described in [74,98] concerning the fact of the concentration of filler particles at the adhesive surface, but also causing the formation of a more brittle material. By comparing these results with the those from Table 7, Table 8 and Table 9, it should be noted that the tensile strength of the samples does not translate directly into adhesion of glue to concrete. The interactions between the adhesive and the concrete substrate are also important. The adhesive layer is relatively thin; therefore, the arrangement of molecules at the surface of the layer, resulting mainly from the theory of adsorption and adsorption theory of adhesion, determines the durability of the joint to the greatest extent [74]. At the same time, as noted in [27,98], it is very important to properly apply the adhesive to a given surface, regardless of the type of its treatment. Mechanical adhesion is also relevant. As noted in [74] in modified resins, the internal structure—which determines the tensile strength of the samples to the greatest extent—may differ from the surface structure, affecting the effectiveness of the gluing process. Therefore, the tensile strength is not the only factor that determines the total adhesion of the adhesive to the substrate. 

Individual test results require a separate analysis in terms of the effectiveness of the modifications applied for each of the prepared surfaces due to different final effects. In the case of a cleaned surface, characterized by the lowest specific surface area and bond activation energy [1,3,96], the durability of the joint is primarily determined by mechanical adhesion, and to a lesser extent by the adsorption theory. The highest value of the pull-off stresses was obtained for the adhesive modified only by sonication, and it was 71% higher than the value for the unmodified adhesive. The ER52/S/M and ER52/S/N series were characterized by a smaller increase, by 24% and 18%, respectively. As an explanation of the phenomena, it was found that the sonified resin, the internal structure of which initially disintegrated due to the occurrence of ultrasonic cavitation, becomes more ordered after hardening, which was also observed in [77]. At the same time, as the research included in [75] has shown, free ends of polymer chains are located near the surface, which more easily bind to the substrate. The series with the addition of fillers are characterized by a smaller increase in adhesion. This is due to the fact that some of the free ends are joined by filler molecules, especially carbon nanotubes. However, according to the adsorption theory, filler molecules can form temporary bonds with the components of the substrate, mainly due to the presence of Van der Waals forces [7,37]. 

The data in Table 8 suggest other relationships. The ground surface, according to the information contained in [90,91], is characterized by a rougher structure, which also can be inferred from the comparison of Figure 7, Figure 8 and Figure 9. This results in a greater tendency to form mechanical adhesion joints. At the same time, due to the partial exposure of aggregate grains, it is possible to activate the surface to a greater extent in terms of the possibility of creating chemical bonds between the adhesive and the substrate [7,97]. This is reflected in the pull-off test results. For the ER52/S and ER52/S/M series, the adhesion to the ground surface was about 20% higher than for the unmodified resin. The largest increase was recorded for the ER52/S/N series and amounted to 70%. When analyzing the obtained results, it should be noted that the obtained adhesion increases occur due to a greater proportion of adsorptive adhesion as well as the possibility of creating chemical bonds between the modified resin particles and the concrete substrate. At the same time, the ER52/S/N series was characterized by the highest value of viscosity in the liquid state (Figure 6). Therefore, from the very beginning, the adhesive bonded more permanently to the substrate before it was completely cured. Importantly, the higher viscosity did not interfere with the possibility of joining with the substrate due to the mechanical adhesion. 

The data in Table 9 allow for drawing further conclusions. For all modified series, there was a clear increase in the adhesion of the samples and amounted to 28% for the ER52/S series, 81% for the ER52/S/M series and 63% for the ER52/S/N series, respectively. As stated also in [3,45,99,100], the main reason may be the occurrence of mechanical and adsorptive adhesion mechanisms to a greater extent than in the case of previously described surfaces. The sandblasted surface has the best-developed specific surface, which is the most diverse, although, as noted earlier, it is not as regular as in the case of the ground surface. At the same time, greater widths of individual grooves and recesses, as well as more diversified profile, allow the adhesive to penetrate to a greater depth. Of course, the viscosity of the adhesives and the modification method are also important. In this case, the relations were similar as in the case of the results obtained for the ground surface. One should also take into account the so-called weak boundary layers which, according to the adhesive theory, may weaken the adhesion between two materials [2,4,20]. The method of surface preparation confirmed the conclusions found in [3,92,93,94], which pertain to the increase of mechanical adhesion along with the unevenness and roughness of the substrate. At the same time, for the series of resins with fillers, apart from mechanical adhesion, the formation of additional chemical bonds between the adhesive and the substrate had a significant impact on the value of pull-off stresses.

**Figure 10:**
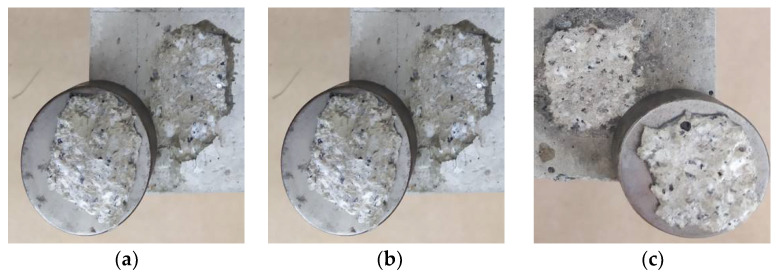
Image of the destruction detached samples for ER52/S series, (**a**) cleaned surface, (**b**) ground surface, (**c**) sandblasted surface.

As an additional analysis, the nature of damage to the samples after detaching from the concrete surfaces was assessed. Figure 10 shows the effect of tests carried out for the ER52/S series. A similar process of destruction was found for each of the tested series. Stick piece of CFRP tape was detached along with the adhesive layer and the sub-surface concrete layer containing the slurry and smaller aggregate grains. This effect was most visible in the case of the ground surface for each series. As described in [99,100], the separation of the reinforcing tape from the material surface may occur as a result of tape delamination (destruction), destruction of the adhesive layer in the adhesive joint or, e.g., in reinforced concrete structures, as a brittle cohesive failure. The last model of destruction results from the separation of the concrete layer (concrete cover) with the tape and the adhesive. The damage to the tape or the adhesive layer itself, as well as intermediate states, was not observed. Grounding ensures the most even distribution of unevenness (Figure 8) and the discovery of the aggregate surface to which adhesive binds. The result of this process is the formation of permanent and temporary chemical bonds. From the point of view of the effectiveness of gluing, it is therefore a favorable phenomenon, which proves that the resins prepared according to the described recipes can penetrate into the unevenness of the substrate correctly. However, a various-load scheme of such a connection [99,101] should also be taken into a account, along with its working conditions and the impact of other environmental factors. In order to strengthen the durability of the adhesive in the joint, apart from the possibility of modification with fillers, it is also possible to additionally improve it mechanically with pins [102] or special grips [103].

### 3.4. Correlation Analysis

Table 11 contains a correlation matrix by means of it is possible to estimate the correlation between individual rheological and mechanical properties. Two correlation coefficients were used in the correlation analysis: Pearson (r) and Spearman (ρ). The first correlation coefficient (Pearson) allows to determine the strength of the linear correlation between individual parameters. The Spearman correlation coefficient determines the strength of each correlation characterized by monotonicity, but the condition of the correlation linearity does not apply in this case. The method and criterion of interpretation of both coefficients is the same, but a careful analysis of the values of the correlation coefficients allows to determine whether the dependence meets the criteria of linear or non-linear correlation.

The global analysis of correlation matrix indicates a more frequent and stronger occurrence of linear than non-linear relationships. It is also worth noting that the method of surface preparation is also of great importance in the overall analysis of the strength of correlation. The strongest correlation was found for HV10 hardness and the pull-off stress for the cleaned surface (|r| = 0.88 and |ρ| = 0.80). A relatively strong linear relationship between the tensile strength value and the pull-off strength for each type of surface was also noted (|r| = 0.55, 0.6 and 0.75). In the case of correlation between viscosity and pull-off stress for the ground surface, the dependence that is present indicates to a greater extent the occurrence of a non-linear relationship than a linear (|r| = 0.30 and |ρ| = 0.98). Moreover, the inverse relationship between the viscosity and the hardness of the surface (|ρ| = 0.6) is also noteworthy. The obtained relationships indicate that the state of the resin in the liquid phase and its ability of filling in the unevenness of the substrate has a diversified influence on its properties in the hardened state. All the processes that take place after applying the adhesive, including the process of hardening the resin and creating chemical and mechanical connections with the elements of the concrete substrate, are important. The remaining dependencies and correlations show a weak or moderate correlation. The analysis also shows that it is possible to efficiently estimate the individual properties of the resin, especially in terms of viscosity, hardness and the mechanical properties of the hardened adhesive on different surfaces. Nevertheless, in the case of the conducted analyses, the lack of a strong relationship in most cases may be as result from the complex phenomenon of adhesion, depending on many factors. All of them can be considered separately in other descriptions, while their total effect on adhesion is complex and requires careful analysis.

## 4. Conclusions

The article presented the results of research on the physico-mechanical and rheological properties of epoxy resins (included in previous studies) modified by means of the sonication process and the addition of a filler in the form of microsilica and carbon nanotubes. A profilometric analysis of the concrete surface, prepared according to three methods, was also performed. In view of the literature analysis as well as the results of the conducted research and their interpretation, the following final conclusions were drawn:

- The differences in the viscosity values of the tested resins resulted directly from the modification method and the type of filler used. This allowed for the assessment of the potential initial adhesion of the glue to the substrate at the time of its application. Modification of the material by means of ultrasounds suggests the possibility of creating more durable bonds at the resin—substrate interface due to a 14% increase in viscosity. The content of microsilica in the amount of 0.5%, due to the binding of some of the free ends of the polymer chains, causes a 13% decrease in viscosity, which may result in greater ability of the adhesive to penetrate into the unevenness of the substrate. Carbon nanotubes in the amount of 0.1%, due to unfolding and breaking chemical bonds between carbon atoms and creating a very large specific surface, as a result of their connection with mers, cause a significant densification of the structure of resins resulting in a threefold increase in viscosity.
- The method of surface preparation significantly influences the mechanism of adhesion formation between the adhesive with which the CFRP tapes were glued and the concrete substrate. Surface treatment not only removes weak boundary layers, but also develops surface roughness. Depending on the chosen method, a more or less even distribution of pits and unevenness of the substrate can be obtained, which has a decisive influence on the mechanical adhesion. The most favorable effect in the form of a developed surface roughness was obtained in the case of sandblasting and grinding.
- The ER52/S, ER52/S/M and ER52/S/N series were characterized by greater adhesion to any type of substrate. For cleaned surface, the highest increase in adhesion by 71% was recorded for the ER52/S series. The ER52/S/M and ER52/S/N series were characterized by a similar increase at the level of 18–24%. The highest adhesion on the ground surface was noted for the ER/S/N series (increase by 70%) and almost the same for the ER52/S/M and ER52/S series, characterized by an increase of 20%. The series tested on the sandblasted surface were characterized by the most diverse changes in adhesion. The largest increase of 81% was recorded for the ER52/S/M series, followed by an increase of 63.5% (ER42/S/M series) and 28% (ER52/S series). The percentage increase in adhesion depended on the method of glue modification and the method of surface preparation, primarily related to the removal of weak boundary layers.
- The correlation analysis carried out predominantly showed the existence of strong non-linear relationships between the mechanical properties.
- Differences in the tensile strength values do not directly translate into the results of the test of adhesion to concrete substrate.
- The obtained test results suggest the possibility of adapting the method of surface modification as well as the described modifications to the conditions under which the adhesive application may take place.

## Figures and Tables

**Table 1:** Mechanical properties of FRP composites.

Type of FRP	Tension Strength [MPa]	Elasticity Modulus [GPa]	Density [g/cm^3^]
GFRP	480–4580	35–86	1.25–2.50
CFRP	600–3920	37–784	1.50–2.10
AFRP	1720–3620	41–175	1.25–1.45
BFRP	600–1500	50–65	1.90–2.10
steel *	280–1900	190–210	7.85

* steel for comparison.

**Table 2:** Properties of the glue used in tests.

Resin	Epidian 52
Form	yellow, dense liquid
Flashpoint [°C]	64
Gelation time [min]	40
Epoxy number [mol/100 g]	0.51–0.55
Density (22 °C) [g/cm^3^]	1.12–1.13
Viscosity (22 °C) [Pa·s]	0.4–0.8
Solubility	ketones, esters, alcohols

**Table 3:** Recipes and series used in research.

Series	Resin Type	Type of Additive/Modification	Amount of Filler [%]	Amount of Hardener [%]
ER52	epoxy	-	-	10
ER52/S		sonication	-	10
ER52/S/M		sonication + microsilica	0.5	10
ER52/S/N		sonication + carbon nanotubes	0.1	10

**Table 4:** Roughness profile parameters for individual surfaces.

Surface	R_p_	R_v_	R_z_	R_c_	R_t_	R_a_	R_q_	R_sm_	R_sk_	R_ku_
**C**	7.04	7.90	14.90	7.20	26.50	2.77	3.46	0.14	−0.11	3.00
**G**	12.60	17.10	29.70	16.00	45.00	5.66	6.96	0.15	−0.42	2.70
**S**	15.50	21.10	36.50	22.60	47.40	6.95	8.74	0.30	−0.47	2.89

**Table 5:** Results of compressive test strength of concrete specimens.

Specimen	Force [kN]	Stress [MPa]	Coefficient of Variation [%]	Average 15 × 15 × 15 cm [MPa]
1	1002.78	44.60	3.1	42.80
2	974.75	43.30		
3	967.35	43.00		
4	924.57	41.10		
5	943.21	41.90		

**Table 6:** The criterion for assigning concrete to a class.

No.	Compression Strength [MPa]	Average Strength f_cm_ [MPa]	Characteristic Strength f_ck_ [MPa]	Criteria 1 f_cm_ ≥ f_ck_ + 4		Criteria 2 f_ci_ ≥ fck − 4			Concrete Class
1	44.60	42.80	37	42.80	≥	41	44.60	≥33	C30/37
2	43.30						43.30		
3	43.00						43.00		
4	41.10						41.10		
5	41.90						41.90		

**Table 7:** Results of pull-off test for the cleaned surface (C).

Series	Force [kN]	Pull-Off Stress [MPa]	Coefficient of Variation [%]
ER52	3.23	4.31	0.70
ER52/S	5.53	7.37	2.60
ER52/S/M	4.01	5.35	3.00
ER52/S/N	3.83	5.10	3.20

**Table 8:** Results of pull-off test for the ground surface (G).

Series	Force [kN]	Pull-Off Stress [MPa]	Coefficient of Variation [%]
ER52	3.15	4.20	1.80
ER52/S	3.79	5.05	2.60
ER52/S/M	3.80	5.07	2.10
ER52/S/N	5.35	7.14	0.60

**Table 9:** Results of pull-off test for the sandblasted surface (S).

Series	Force [kN]	Pull-Off Stress [MPa]	Coefficient of Variation [%]
ER52	3.61	4.82	1.90
ER52/S	4.62	6.17	0.60
ER52/S/M	6.55	8.73	2.40
ER52/S/N	5.89	7.85	2.30

**Table 10:** Results of tensile strength of resins and surface hardness [77].

Series	ER52	ER52/S	ER52/S/M	ER52/S/N
Hardness HV10	20.35	21.40	20.60	21.03
Tensile strength [MPa]	36.97	42.53	29.67	29.44

**Table 11:** Matrix of Pearson and Spearman correlation coefficients.

Correlation		Pearson’s Correlations (r)					
	Value	Viscosity	HV10	Tensile Strength	Pull-Off C	Pull-Off G	Pull-Off S
**Spearman’s correlations (ρ)**	**Viscosity**	-	0.34	−0.46	−0.15	0.30	0.30
	**HV10**	0.60	-	0.40	0.88	0.46	0.14
	**Tensile strength**	−0.20	0.20	-	0.60	−0.55	−0.75
	**Pull-off C**	0.20	0.80	0.40	-	0.03	0.07
	**Pull-off G**	0.98	0.40	−0.80	0.20	-	0.58
	**Pull-off S**	−0.20	0.20	0.20	0.40	0.80	-

Designation of correlation strength: |r|<0.2—weak correlation; |r|∈〈0.2÷0.4)—low correlation; |r|∈〈0.4÷0.6)—moderate correlation; |r|∈〈0.6÷0.8)—high correlation; |r|∈〈0.8÷0.9)—very high correlation; |r|∈〈0.9−1.0〉—correlation virtually complete.
